# *Notes from the Field*: Retrospective Analysis of Wild-Type Measles Virus in Wastewater During a Measles Outbreak — Oregon, March 24–September 22, 2024

**DOI:** 10.15585/mmwr.mm7502a1

**Published:** 2026-01-15

**Authors:** Rebecca Falender, Melissa Sutton, Paul Cieslak, Juventila Liko, David Mickle, Christine Kelly, Tyler Radniecki

**Affiliations:** ^1^Oregon State University, Corvallis, Oregon; ^2^Oregon Health Authority, Portland, Oregon.

**Summary**

**What is already known about this topic?**

Wastewater surveillance can be used to monitor emerging pathogens, including wild-type measles virus.

**What is added by this report?**

During a June 11–September 26, 2024, measles outbreak in Oregon, which included a close-knit community that did not readily seek health care, a retrospective analysis of archived regional wastewater data collected during March 24–September 22, 2024, detected wild-type measles virus in 20 of 94 (21.3%) samples. The first detection of measles virus in wastewater was in a sample collected on April 3, 2024, which preceded the first confirmed measles case by 10 weeks.

**What are the implications for public health practice?**

Wastewater surveillance can provide an early warning of community measles circulation and can guide the public health response during outbreaks, including recommendations for vaccination.

In 2024, Oregon reported 31 measles cases in residents of three counties, the highest case count in Oregon since 1991. Thirty of these cases were associated with an outbreak in Clackamas and Marion counties, which included a close-knit community that did not readily seek health care; all cases occurred in unvaccinated persons. Illness onset for the person with the first confirmed case occurred on June 11, 2024, and the outbreak was declared over approximately 15 weeks later on September 26, a total of 42 days after illness onset in the last person with measles. Wastewater surveillance is a useful tool in the surveillance of emerging pathogens, including avian influenza A(H5) (*1*); however, wastewater surveillance for measles virus has not been well described in the context of clinical data. This retrospective study describes the detection of wild-type measles virus in wastewater samples collected during the 2024 measles outbreak.

## Investigation and Outcomes

### Data Collection

Oregon began wastewater surveillance for SARS-CoV-2 (the virus that causes COVID-19) in 2020. Routine wastewater surveillance now includes SARS-CoV-2, influenza, respiratory syncytial virus, influenza A(H5), and, since October 2025, wild-type measles virus. Unlike vaccine-derived measles virus, wild-type measles virus is transmitted from person to person and can cause outbreaks. Wastewater surveillance activities include collecting and archiving 24-hour composite samples from up to 40 wastewater treatment facility influents statewide once or twice weekly (*1*). To ascertain the presence of wild-type measles virus in wastewater in the outbreak-affected area, archived specimens collected during March 19–September 26, 2024, were retrospectively tested during July and August 2025 from four communities in the two counties with outbreak cases. The study period initially ranged from April 30 through September 26, which was two incubation periods (42 days) before illness onset for the first case through two incubation periods after onset for the last case. When wild-type measles virus was detected in samples from the first week of the study period (on May 5, 2024), the beginning of the study period was extended to include four incubation periods before illness onset for the first case (i.e., March 19–September 26) (*2*).

### Processing of Samples

Filtered, preserved 24-hour composite wastewater influent samples were homogenized using bead-beating with 0.7-mm garnet beads to lyse the cells. Nucleic acids were extracted from 200–400 *μ*l of the homogenate and analyzed for a wild-type–specific measles virus target using reverse transcription–digital polymerase chain reaction (RT-dPCR) (*1*,*3*). Detections were defined as samples with a viral concentration above the assay limit of detection, which was calculated based on the assay limit of blank.^†^ Data were analyzed in RStudio (version 4.3.1; RStudio, Inc.). This activity was reviewed by the Oregon Health Authority, deemed not research, and was conducted consistent with federal law.^§^

### Detections of Wild-Type Measles Virus in Wastewater

Among 94 analyzed samples collected during March 19–September 26, 2024, a total of 20 (21.3%) tested positive for wild-type measles virus (Figure). The first detection of measles virus in wastewater was in a sample collected on April 3. Wastewater detections preceded reported cases by approximately 10 weeks. After scattered detections of measles virus at low concentrations (i.e., above the limit of detection but fewer than three positive dPCR partitions) in both counties, a period during which measles virus was detected at higher concentrations (i.e., above the limit of detection and three or more positive dPCR partitions) occurred during June 12–July 23, corresponding with the first reported clinical cases. The last sample in which measles virus was detected was collected on July 24. Overall, 11 (55%) of the 20 measles virus detections were in samples collected during the outbreak period. No virus was detected in wastewater during the last 9 weeks of the outbreak. After the last detection of wild-type measles virus in a wastewater sample, an additional eight cases were reported.

**FIGURE:**
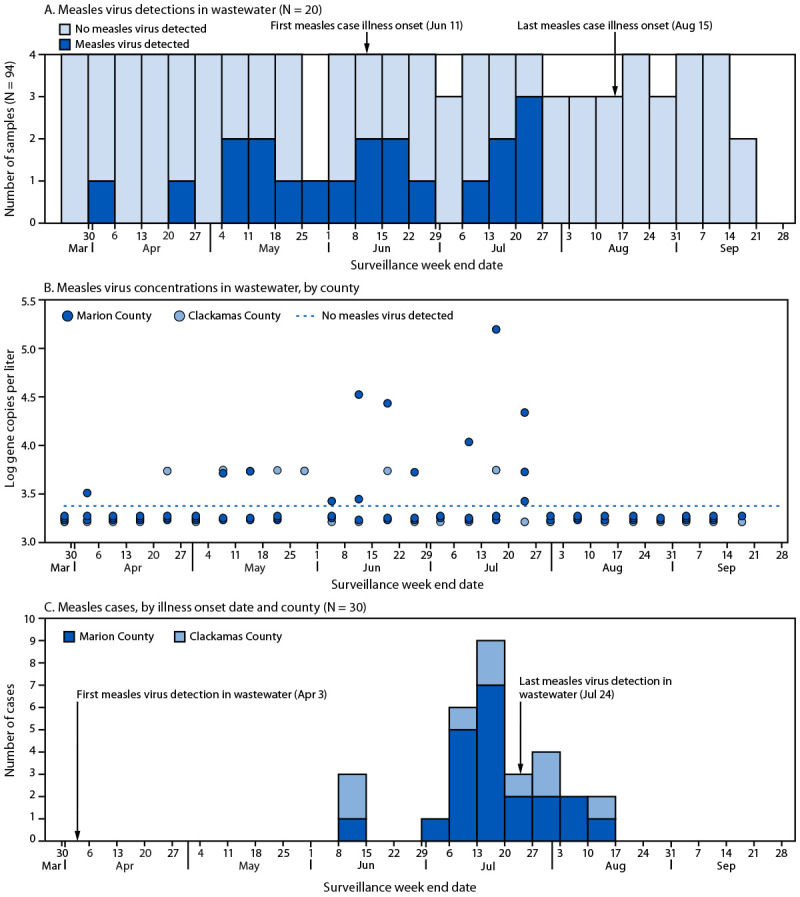
Wild-type measles virus detections (A) and concentrations* (B) in wastewater and confirmed and probable measles cases (C), by surveillance week end date — Clackamas and Marion counties, Oregon, March–September 2024 * Samples below the nondetection line were negative for measles virus. Jitter (slight displacement of data points from overlapping and obscuring one another) was applied to all nondetection and overlapping detection data points.

## Preliminary Conclusions and Actions

Wastewater surveillance can provide an early warning signal for emerging pathogens, including measles, independent of health care–seeking behavior and access to testing (*1*). In this retrospective study, wastewater detection of wild-type measles virus preceded the first reported case by 10 weeks. A 6-week period of higher concentrations of measles virus in samples corresponded to the outbreak peak.

The findings in this study are subject to at least two limitations. First, because the measles viruses detected in the wastewater samples were not sequenced, whether all detections were epidemiologically linked to the outbreak is unknown. Second, the absence of measles virus detections in wastewater samples does not rule out the presence of measles in a community, as evidenced by identification of eight cases after the last wastewater sample tested positive for measles virus.

During 2025, the United States experienced the highest number of measles cases since elimination was declared in 2000. To prevent transmission, systems to rapidly identify, isolate, and investigate suspected measles cases, as well as high population immunity, are needed (*4*). This study highlights the usefulness of wastewater surveillance as an early warning of measles in a community, with the potential to detect community transmission before the first cases have been identified. Wastewater surveillance can alert clinicians and the public to a current measles risk in the community, guide health care system screening procedures and testing practices, and direct important individual-level protective behaviors, including vaccination.

## Footnotes

† A total of 40 no template control (NTC) samples were used to calculate the assay limit of blank (LOB). Assay LOB = (Average gene copies per *μ*l of reaction of NTCs) + 1.654 × (Standard deviation of gene copies per *μ*l of reaction of NTCs). Assay limit of detection (LOD) = Assay LOB × 3 × (Standard deviation of gene copies per *μ*l of reaction of NTCs). Process LOD accounts for volume of wastewater filtered. Process LOD = Assay LOD × (*μ*l of reaction/*μ*l of template) × (*μ*l of elution buffer eluted/*μ*l of lysate used for extraction) × (*μ*l of Zymo DNA/RNA Shield used to stabilize and lyse the sample/mL of wastewater filtered) × (1,000 mL of wastewater/1 L of wastewater).

§ 45 C.F.R. part 46.102(l)(2), 21 C.F.R. part 56; 42 U.S.C. Sect. 241(d); 5 U.S.C. Sect. 552a; 44 U.S.C. Sect. 3501 et seq.
